# Development of Peritoneal Tumor-Targeting Vector by *In Vivo* Screening with a Random Peptide-Displaying Adenovirus Library

**DOI:** 10.1371/journal.pone.0045550

**Published:** 2012-09-20

**Authors:** Takeshi Nishimoto, Yuki Yamamoto, Kimiko Yoshida, Naoko Goto, Shumpei Ohnami, Kazunori Aoki

**Affiliations:** 1 Division of Gene and Immune Medicine, National Cancer Center Research Institute, Chuo-ku, Tokyo, Japan; 2 Central Radioisotope Division, National Cancer Center Research Institute, Chuo-ku, Tokyo, Japan; 3 Department of Neurosurgery, Graduate School of Medicine, Hiroshima University, Minami-ku, Hiroshima, Japan; University of Chicago, United States of America

## Abstract

The targeting of gene transfer at the cell-entry level is one of the most attractive challenges in vector development. However, attempts to redirect adenovirus vectors to alternative receptors by engineering the capsid-coding region have shown limited success, because the proper targeting ligands on the cells of interest are generally unknown. To overcome this limitation, we have constructed a random peptide library displayed on the adenoviral fiber knob, and have successfully selected targeted vectors by screening the library on cancer cell lines *in vitro*. The infection of targeted vectors was considered to be mediated by specific receptors on target cells. However, the expression levels and kinds of cell surface receptors may be substantially different between *in vitro* culture and *in vivo* tumor tissue. Here, we screened the peptide display-adenovirus library in the peritoneal dissemination model of AsPC-1 pancreatic cancer cells. The vector displaying a selected peptide (PFWSGAV) showed higher infectivity in the AsPC-1 peritoneal tumors but not in organs and other peritoneal tumors as compared with a non-targeted vector. Furthermore, the infectivity of the PFWSGAV-displaying vector for AsPC-1 peritoneal tumors was significantly higher than that of a vector displaying a peptide selected by *in vitro* screening, indicating the usefulness of *in vivo* screening in exploring the targeting vectors. This vector-screening system can facilitate the development of targeted adenovirus vectors for a variety of applications in medicine.

## Introduction

Recombinant adenovirus vectors have been widely used for gene delivery to a range of cell types and employed in a number of gene therapy approaches [1]. On the other hand, a selective delivery of a therapeutic gene to target cells by adenovirus vectors is precluded by the widespread distribution of the primary cellular receptors for adenoviruses [2], [3]. The suppression of naïve viral tropism is needed to reduce the undesirable infection of non-target normal tissues, whereas the antitumor effect of adenovirus vector is determined by the capacity to infect tumor cells. Thus, the addition of a tumor-targeting potential to an adenovirus vector ablated for naïve tropism is important to enhance its therapeutic index.

Recently, several strategies have been developed to redirect the tropism of the adenovirus vector to permit efficient target gene delivery to specific cell types [4], [5]. In particular, targeting has been achieved by direct genetic modifications of the capsid proteins: targeting ligands can be incorporated into the C-terminal and HI-loop of fiber proteins ablated for native tropism, and these vectors provide an important platform for evaluating the targeting potential of selected peptide ligands [6]–[9]. However, cell-type specific ligands for targeted adenovirus vectors are generally unknown, which impedes the wider application of fiber-modified adenovirus vectors for targeted therapies. Although a phage display library has been used to identify targeting peptide motifs, the incorporation of the peptides selected by phage display into the adenoviral capsid has not been successful in developing targeted vectors except for a few cases [10]–[12], possibly due to the peptide-induced conformational change of the virus capsid and the loss of specificity and affinity of ligand-receptor binding [13]. To overcome this limitation, we have developed a system for producing adenoviral libraries displaying a variety of peptides on the fiber by Cre-lox mediated *in vitro* recombination between an adenoviral fiber-modified plasmid library and an adenoviral DNA-terminal protein complex, and have established a procedure to select an adenoviral vector with high infectivity in target cells *in vitro* [14], [15]. Another group also reported a method for generating libraries displayed on an adenovirus fiber in which modified-fiber genes were directly shuttled into a replicating virus genome in helper cells [16]. Since the binding affinity might be determined by the overall conformation of a modified fiber and not by inserted peptides alone, the selection of targeting peptides is highly useful in the context of the adenoviral capsid.

We were able to successfully screen adenovirus vectors displaying targeted peptide sequences after several rounds of amplification of viruses on cultured cancer cell lines [14], [15]. The infection of targeted vectors was considered to be mediated by specific receptors on target cells. However, the expression levels and kinds of cell surface receptors may be substantially different between *in vitro* culture and *in vivo* tumor tissue. Therefore, an *in vivo* screening of the peptide display adenovirus library may be useful to develop vectors which specifically transduce certain tumors *in vivo*. To this aim, here we examined whether the tumor-targeting vectors could be selected from the adenovirus library by *in vivo* screening in a murine peritoneal dissemination model of pancreatic cancer cells. A particular sequence was observed after 2 rounds of selection, and the adenovirus displaying the selected peptide showed a high infectivity preferentially for peritoneal tumors but not organs. The results demonstrated that an *in vivo* screening with an adenovirus library is a promising strategy for the development of targeted vectors, for which this is the first report of a targeted adenovirus developed by an *in vivo* approach alone.

## Materials and Methods

### Cell Lines

A human embryonic kidney cell line (293), pancreatic cancer cell lines (AsPC-1 and PSN-1), gastric cancer cell line (MKN45) and ovarian cancer cell line (SKOV3) were used in this study. All the cancer cell lines except for PSN-1 were obtained from American Tissue Culture Collection (ATCC; Rockville, MD), and the PSN-1 cell line was established by H. Yamada et al. [17]. 293 cells were cultured in Dulbecco’s modified eagle’s medium (Sigma, St. Louis, MO) with 10% fetal bovine serum (FBS), and cancer cell lines in an RPMI-1640 medium (Nissui Pharmaceutical, Tokyo, Japan) with 10% FBS. 293.HissFv.rec cells express an artificial receptor against six histidine (His) residues, containing an anti-His single chain antibody (sFv) [9]. The 293-38 is a high-efficiency virus-producing clone of 293 cells, and the 293-38 cells expressing an anti-His sFv stably (293-38.HissFv.rec) were generated by retrovirus-mediated transduction [14].

### Shuttle Plasmids and Recombinant Adenovirus DNA

The adenoviral shuttle plasmids pBHI and pBHIΔCAR include a 76.1–100 map unit (mu) of the type 5 adenoviral genome with a single loxP site at the E3 region deleted (79.4–84.8 mu) [14], [15]. The pBHI has a wild type of fiber. The pBHIΔCAR has two incompatible restriction enzyme sites in the HI-loop to display random peptides and includes 4-point mutations in the AB-loop of the fiber knob to abolish CAR binding, and six histidine residues were incorporated into the carboxy-terminal of the fiber knob, so that the vector can be propagated in the 293 cells expressing an anti-His sFv [9]. The pBHIΔCAR-PFW and pBHIΔCAR-SYE plasmids have CCTTTTTGGAGTGGGGCTGTT (PFWSGAV) and TCGTATGAGAATTTTAGTGCG (SYENSFA) sequences in the HI-loop of the pBHIΔCAR plasmid, respectively. The adenoviral cosmid cAd-WT includes the 0–79.4 mu of the adenovirus genome containing a wild-type E1 region and a single loxP site at 79.4 mu. The cAd-WT was recombined with pBHIΔCAR to generate AdΔCAR-WT for preparation of adenoviral DNA tagged with a terminal protein (DNA-TPC). In the cAd-LucEGFP, the E1 gene is replaced by the CMV promoter-driven luciferase-EGFP fusion gene (LucEGFP) in cAd-WT. The cAd-LucEGFP was recombined with pBHIΔCAR-PFW, pBHIΔCAR-SYE, pBHIΔCAR and pBHI plasmids to generate adenovirus vectors AdΔCAR-LucEGFP-PFW, AdΔCAR-LucEGFP-SYE, AdΔCAR-LucEGFP and Ad-LucEGFP, respectively. The adenovirus vectors were quantified by optical absorbance [18]. The infectious units of the viruses were examined in 293-38.HissFv.rec cells, and the ratio of the viral particle to infectious unit for each virus was approximately 30.

### Construction of a Random Peptide-display Adenovirus Library

The random peptide-display adenovirus library used in this study was the same as we previously screened on AsPC-1 cells *in vitro* [15]. We used the adenovirus vector ablated for CAR binding as a backbone construct of adenovirus library to reduce natural tropism. Briefly, the adenovirus shuttle plasmid pBHIΔCAR-EGFP has a cytomegalovirus immediate early enhancer/promoter (CMV promoter), the enhanced green fluorescent protein (EGFP) gene and a SV40 poly (A) signal downstream of the loxP site at the deleted E3 region in the pBHIΔCAR plasmid. The degenerate oligonucleotide 5′-AACGGTACACAGGAAACAGGAGACACAACTTTCGAA(NNK)_7_ACTAGTCCAAGTGCATACTCTATGTCATTTTCATGG-3′ (N = A, T, G or C, K = G or T) served as a template for PCR with the primers 5′-GAAACAGGAGACACAACTTTCGAA-3′ and 5′-CATAGAGTATGCACTTGGACTAGT-3′. The PCR product was ligated into the HI-loop portion of pBHIΔCAR-EGFP to construct a fiber-modified shuttle plasmid library, and transfected into Max Efficiency electrocompetent cells (Invitrogen, Carlsbad, CA) by electroporation. The fiber-modified shuttle plasmid library was recombined with equal moles of the left hand of the digested DNA-TPC by Cre recombinase (Clontech, Madison, WI) *in vitro* to produce a full-length adenovirus genomic DNA library. Then, to generate replication-competent peptide-display adenovirus libraries, recombined adenoviral DNA was transfected by the lipofection method (Lipofectamine Reagent; Invitrogen) in 293-38.HissFv.rec cells. When the cells showed an expansion of the cytopathic effect, the first generation of the adenovirus library was harvested. 293-38.HissFv.rec cells were infected with the crude viral lysate (CVL) again, and the second generation of the library was harvested. The library was estimated to display more than 1×10^4^ peptides on the fiber per 60-mm dish [14]. Since the library used in the screening was collected from twenty 60-mm dishes, the complexity of the peptide sequences displayed in the library was estimated to be approximately at a 2×10^5^ level, and the final concentration of the virus library was prepared as 1×10^9^ plaque forming unit (PFU)/ml.

### Screening of a Random Peptide-display Adenovirus Library in Mice with Peritoneal Dissemination

Four to 5-week-old female BALB/c nude mice were purchased from Charles River Japan, Inc. (Kanagawa, Japan), and were housed under sterilized conditions. Animal studies were carried out according to the Guideline for Animal Experiments of the National Cancer Center Research Institute and approved by the Institutional Committee for Ethics in Animal Experimentation. AsPC-1 (5×10^6^) cells were intraperitoneally injected into the mice, which resulted in the peritoneal dissemination and the formation of tumor nodules in pancreatic regions within 14 days. Eighteen days after the injection of tumor cells, 200 µl of an adenovirus library solution (1×10^8^ PFU) was intraperitoneally injected into the mice. Seven days following the injection, peritoneal tumors were harvested, and after the pulverization of the tumors, the CVL was prepared from the tumors by freezing and thawing 3 times. To expand the selected viruses, 293-38.HissFv.rec cells were infected with the CVL from the peritoneal tumors. Seven days after the infection, the CVL from 293-38.HissFv.rec cells was intraperitoneally reapplied in the mice with peritoneal tumors of AsPC-1 cells as a second round of selection. Seven days later the replicated adenoviruses were harvested from the peritoneal tumors again.

### PCR and Sequencing of Adenovirus Library Clones

DNA was extracted from the CVL of the peritoneal tumors by the 1st and 2nd selections, and then served as a template for a PCR with the primers containing upstream and downstream sequences of the HI-loop: 5′-GAAACAGGAGACACAACTTTCGAA-3′ and 5′-CATAGAGTATGCACTTGGACTAGT-3′. PCR products were cloned into the pBHIΔCAR plasmid. Randomly assigned clones were sequenced using the primer 5′- GGAGATCTTACTGAAGGCACAGCC-3′.

### Assay for Luciferase Activity *in vitro*

The cells were seeded at 1×10^4^ per well in 96-well plates (Optilux multiplate; BD Biosciences, Franklin Lakes, NJ) and infected with adenoviruses at various moi (1, 3, 10, 30 and 100). Twenty-four hours after the infection, 100 µl of luciferase assay substrate (Bright-Glo™ luciferase assay system; Promega, Madison, WI) was added to each well. The light units of luciferase activity were measured using a luminometer (EnVision multilabel plate reader; Parkin Elmer, Shelton, CT). The assays (carried out in 8 wells) were repeated a minimum of two times and the mean ± standard deviation was plotted.

### Assay for Luciferase Activity *in vivo*

To examine the *in vivo* infectivity of a targeted adenovirus vector, AsPC-1 cells (5×10^6^ cells) were injected intraperitoneally into the BALB/c nude mice. Fourteen days later, 200 µl of a viral solution (1×10^7^, 3×10^7^ and 1×10^8^ PFU) of AdΔCAR-LucEGFP-PFW was intraperitoneally injected into the mice with a 29-gauge hypodermic needle. The luciferase assay was performed as described previously [19].

### Detection of Adenovirus DNA from the Peritoneal Tumors and Organs

The peritoneal tumors and organs such as the liver, spleen, pancreas and small intestine were collected 2 days after the intraperitoneal injection of an adenovirus solution (1×10^8^ PFU), and DNA was extracted from the tumors and organs using Sepagene (Sanko Junyaku Co. Ltd., Tokyo, Japan). The viral DNA in 1 µg total DNA was analyzed by a real-time PCR using Eco™ Real-Time PCR system (Illumina Inc., San Diego, CA). The primers to detect a 68-bp region in E4 were utilized as described previously [20]. Briefly, the sequences of the upstream and the downstream E4 primers were 5′-GGAGTGCGCCGAGACAAC-3′ and 5′-ACTACGTCCGGCGTTCCAT-3′, respectively. The sequence of the TaqMan probe (6-FAM-labeled probe) was TGGCATGACACTACGACCAACACGATCT. A final volume of 10 µl/reaction containing 1×Gene Expression Master Mix (Applied Biosystems, Foster City, CA), 100 nM upstream primer, 100 nM downstream primer, 1.1 µM probe and extracted DNA was applied to the real-time PCR. For the standard curve to quantify the E4 copy numbers, E4 template DNA with a known copy number (2.4×10^6^–2.4×10) was also analyzed. Thermal cycling conditions were as follows: initial denaturation at 95°C for 10 min, and then 40 cycles at 95°C for 15 s and at 60°C for 1 min.

### Statistical Analysis

Comparative analysis of luciferase activity was performed by the Student’s t-test, and differences were considered statistically significant when the P value was <0.05.

## Results

### *In vivo* Selection of Library Clones Targeting Peritoneal Tumor

**Figure 1:**
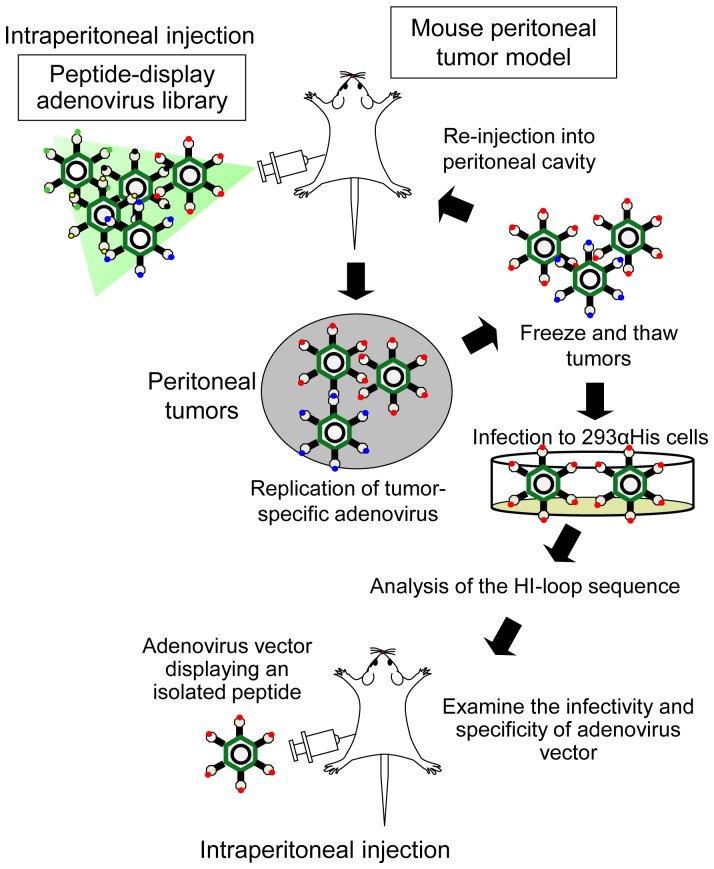
Screening of a random peptide display adenovirus library in mice with peritoneal dissemination. To explore the peritoneal tumor-targeting vector, the adenovirus display library was intraperitoneally injected into the mice with peritoneal dissemination of AsPC-1 cells. Seven days after the injection, the peritoneal tumors were harvested and adenoviruses were recovered from the peritoneal tumors. The adenoviruses expanded on 293-38.HissFv.rec were subjected to a second round of selection. The oligonucleotide sequences in the HI-loop of adenoviruses that proliferated in peritoneal tumors were then analyzed. Less than 4 weeks were required to perform 2 rounds of selection and determine the targeted sequences.

To examine whether an *in vivo* screening could select peptides displayed on the fiber knob that produces higher transduction efficiency to peritoneal tumors, the adenovirus display library was intraperitoneally injected into the mice with peritoneal dissemination of AsPC-1 cells (Fig. 1). In the initial phase of the screening, many low-affinity or nonspecific viruses might bind and internalize into peritoneal tumor cells; however, the use of a replication-competent type of adenovirus could allow for the rapid amplification and spreading of the most efficient viruses present in the library, leading to an effective enrichment of such viruses. Amplified adenoviruses in peritoneal tumors were recovered and subjected to a second round of selection. The DNA region containing the oligonucleotide insert of adenoviruses recovered from the first and second rounds of selection was then amplified by PCR.

DNA sequencing of the PCR products revealed enrichment of various peptides after the first round of selection, whereas 31 of 32 clones (96.9%) showed the same sequence PFWSGAV after the second round of selection (Table 1). The PFWSGAV sequence was recognized in 2 of 41 clones (4.9%) at the first round. The screening was repeated to confirm the reproducibility of the results, and the 1st screening also enriched the same PFWSGAV motif (8 of 32 clones; 25.0%) in peritoneal tumors from the same library. None of the selected sequences was found in the 100 randomly isolated clones from the unselected adenovirus library. The fact that the identical sequence was repeatedly enriched in the 2 independent screenings potentially indicates the feasibility and reliability of an *in vivo* screening procedure to select targeted vectors to tumors. The selected motif PFWSGAV was different from a sequence (SYENFSA) selected by *in vitro* screening on AsPC-1 cells with the same adenovirus library [15]. The peptide motifs selected from the same library may be different according to the environment in which tumor cells grow. To prove that the selected peptides are a result of binding-mediated selection and not due to a low complexity bias in the library, PSN-1 peritoneal tumors were also screened using the same library. All peptide sequences selected from the library after 2 rounds of screenings in PSN-1 peritoneal tumors were different from those selected from AsPC-1 peritoneal tumors (data not shown).

**Table 1:** Peptide sequences selected from a peptide-display adenovirus library in peritoneal tumors of AsPC-1 cells.

	1st. round			2nd. round	
LQTSLGC(5)	ASIGFLV(1)	LQASLGC(1)	PFWSGAV	PFWSGAV	PFWSGAV
CWVRECR(3)	GASVFPT(1)	WLTSSLN(1)	PFWSGAV	PFWSGAV	PFWSGAV
LGTVCDL(3)	WGTVCGL(1)	YMVIWLG(1	PFWSGAV	PFWSGAV	PFWSGAV
RLDGGAV(2)	GVVWYMA(1)	IRQLRFG(1)	PFWSGAV	PFWSGAV	PFWSGAV
GVQWLNG(2)	GGGRGFA(1)	VRLRGGE(1)	PFWSGAV	PFWSGAV	PFWSGAV
ARGSGVC(2)	TECVPPG(1)	VPVVWWR(1)	PFWSGAV	PFWSGAV	PFWSGAV
WLGWAVS(2)	VLSRVCY(1)	FWRCGVD(1)	PFWSGAV	PFWSGAV	PFWSGAV
PFWSGAV(2)	FCNGRAF(1)	SVGCFLG(1)	PFWSGAV	PFWSGAV	PFWSGAV
WVVVFSF(1)	GSVVGRY(1)	EGWVGCD(1)	PFWSGAV	PFWSGAV	PFWSGAV
GARTVVR(1)	TSYGAYC(1)	*G*LEHS(1)	PFWSGAV	PFWSGAV	PFWSGAV
	/42		PFWSGAV	PFWSGAV	VRLRGGE
			PFWSGAV	PFWSGAV	
				/32	

(): Numbers of repetition.

* : Stop codon.

### Infectivity of Adenovirus Displaying a Selected Peptide for AsPC-1 Peritoneal Tumors

**Figure 2:**
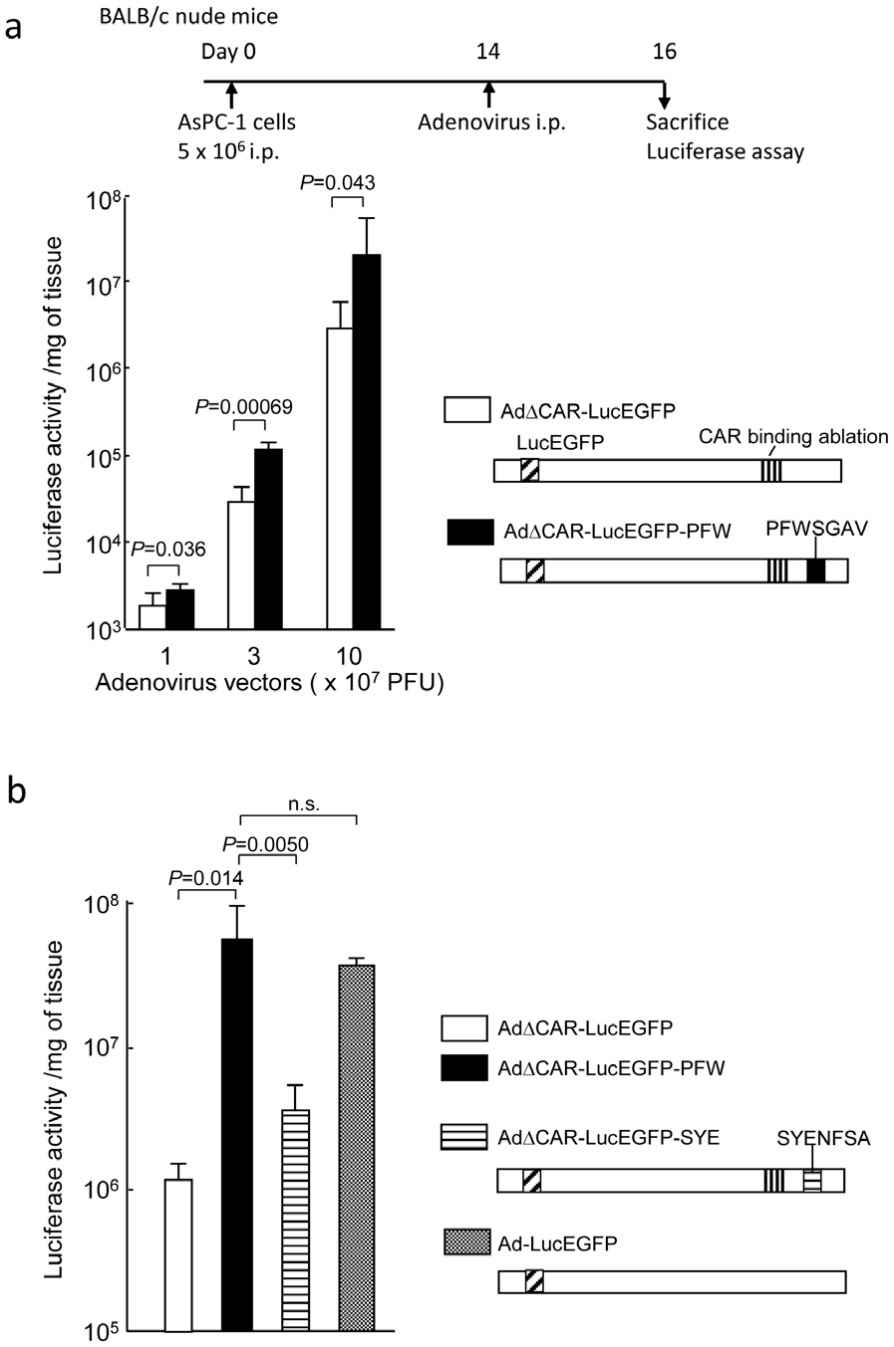
Infectivity of AsPC-1 peritoneal tumors with the adenovirus displaying the selected peptide. (a) Infectivity of AsPC-1 peritoneal tumors with AdΔCAR-LucEGFP-PFW. The various amounts (1, 3, 10×10^7^ PFU) of adenovirus vectors (AdΔCAR-LucEGFP-PFW or AdΔCAR-LucEGFP) were intraperitoneally injected once into the mice with peritoneal dissemination of AsPC-1 cells (n = 3∼4). Two days after the injection, peritoneal tumors were harvested and the luciferase activity was measured. The graph presents the luciferase activity per mg of tumor tissue. AdΔCAR-LucEGFP is a replication-incompetent adenovirus vector ablated for CAR binding and expresses a fusion gene of luciferase and EGFP. In AdΔCAR-LucEGFP-PFW, a PFWSGAV peptide is inserted in HI-loop of fiber knob in AdΔCAR-LucEGFP. i.p.; intraperitoneal injection. (b) Infectivity of AsPC-1 peritoneal tumors with various adenovirus vectors. The 1×10^8^ PFU of adenovirus vectors (AdΔCAR-LucEGFP-PFW, AdΔCAR-LucEGFP-SYE, AdΔCAR-LucEGFP or Ad-LucEGFP) were intraperitoneally injected once into the mice with peritoneal dissemination of AsPC-1 cells (n = 5). Two days after the injection, peritoneal tumors were harvested and the luciferase activity was measured. In AdΔCAR-LucEGFP-SYE, a SYENFSA peptide is inserted in HI-loop of fiber knob in AdΔCAR-LucEGFP. Ad-LucEGFP is a replication-incompetent adenovirus vector with a wild type of fiber, and expresses a fusion gene of luciferase and EGFP. n.s.; not significant.

To test the infectivity of the adenovirus vector displaying a selected peptide, various amounts of a luciferase-expressing targeted vector ablated for CAR binding (AdΔCAR-LucEGFP-PFW) were intraperitoneally injected into the mice with peritoneal dissemination of AsPC-1 cells. The peritoneal tumors were harvested 2 days after the injection, and the luciferase activity in the tumors was measured. In this assay, the replication-incompetent adenoviruses were employed to eliminate the possibility that the high luciferase activity was due to the efficient replication of the adenovirus in the tumors. The luciferase activity assay showed that the gene transduction efficiency of a targeted vector was 1.6-fold higher at 1×10^7^ PFU level, 4.1-fold higher at 3×10^7^ PFU level and 6.9-fold higher at 1×10^8^ PFU level than that of an untargeted adenovirus vector ablated for CAR binding (AdΔCAR-LucEGFP) for AsPC-1 peritoneal tumors (Fig. 2a), indicating the selected peptide significantly enhanced the adenovirus infectivity for target tumors.

To compare the infectivity of AdΔCAR-LucEGFP-PFW with AdΔCAR-LucEGFP-SYE and Ad-LucEGFP, which has a wild type of fiber, in AsPC-1 peritoneal tumors, 1×10^8^ PFU of adenovirus vectors were intraperioneally injected into the mice with AsPC-1 peritoneal dissemination, and peritoneal tumors were harvested 2 days after the injection. The luciferase assay showed that the infectivity of AdΔCAR-LucEGFP-PFW was significantly higher than AdΔCAR-LucEGFP-SYE for AsPC-1 peritoneal tumors (Fig. 2b). Although the fiber knob of AdΔCAR-LucEGFP-PFW was ablated for CAR binding, its infectivity in AsPC-1 peritoneal tumors was compatible with Ad-LucEGFP (Fig. 2b), demonstrating the high infectivity of the adenovirus vector displaying PFWSGAV for AsPC-1 tumors.

**Figure 3:**
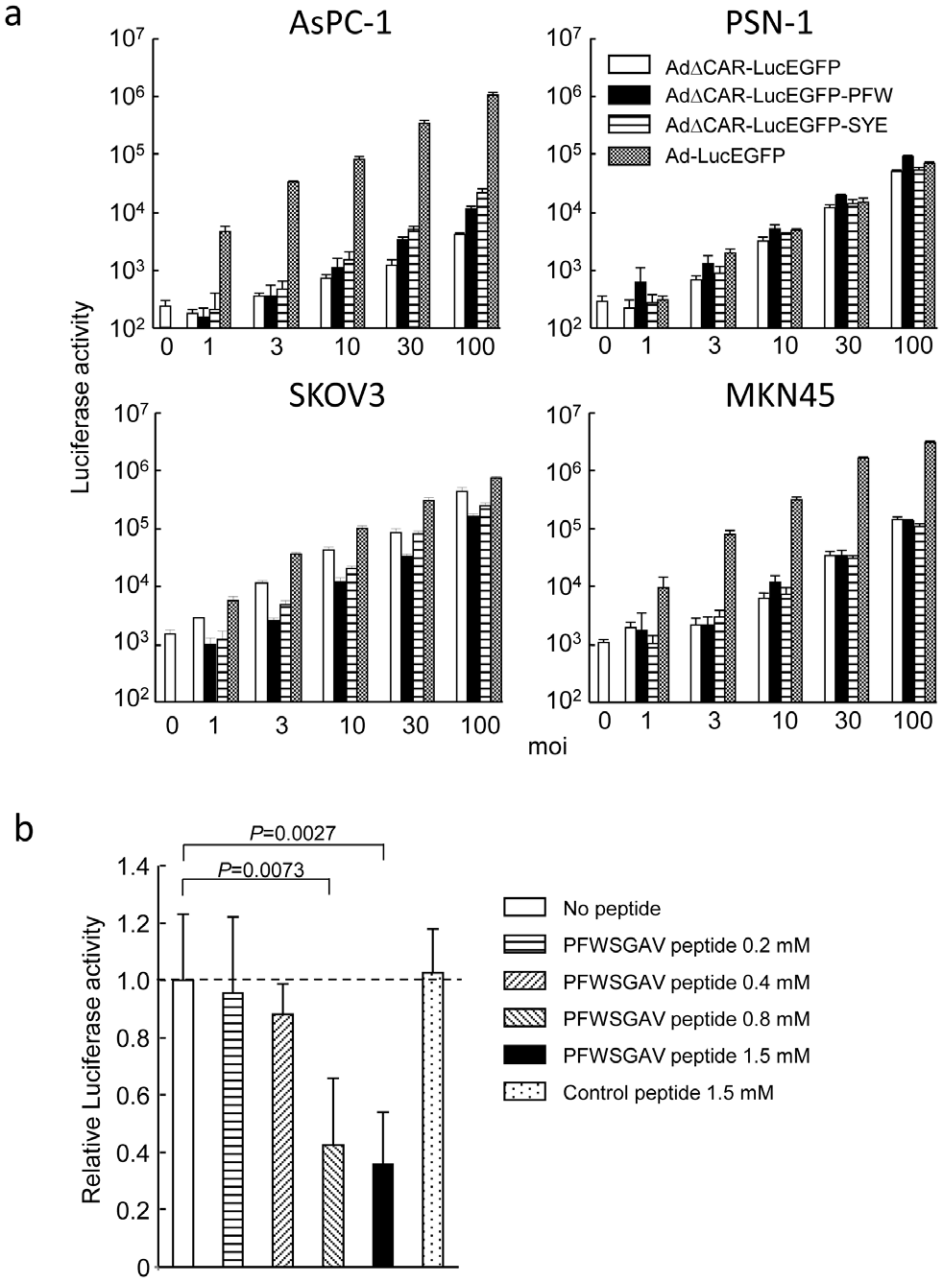
Infectivity of cancer cells with the adenovirus displaying the selected peptide. (a) Luciferase activities following infection of adenovirus vectors *in vitro*. The AsPC-1, PSN-1, SKOV3 and MKN45 cells were infected with adenovirus vectors (AdΔCAR-LucEGFP-PFW, AdΔCAR-LucEGFP-SYE, AdΔCAR-LucEGFP or Ad-LucEGFP), and 24 h later the luciferase activities were measured. (b) Competitive inhibition of transduction of the adenovirus vector with selected peptide. Transduction efficiencies of AdΔCAR-LucEGFP-PFW in AsPC-1 cells were evaluated at moi 30 in the presence of the cognate or a control unrelated peptide at 0.2–1.5 mmol/L. The data are expressed as the relative luciferase activity (luciferase activity in the presence of peptide/that in the absence of peptide). Control peptide: AQGQWAL.

Next, to examine whether gene transduction is also enhanced by the selected peptide *in vitro*, several cancer cells were infected with AdΔCAR-LucEGFP-PFW at various moi. The gene transduction efficiency of AdΔCAR-LucEGFP-PFW was higher in AsPC-1 (2.5-fold at moi 100) and PSN-1 cells (1.8-fold at moi 100) compared with AdΔCAR-LucEGFP, whereas the luciferase activity of AdΔCAR-LucEGFP-PFW-infected cells was lower than those of AdΔCAR-LucEGFP infection in SKOV-3 (0.35-fold at moi 100) and MKN45 cells (0.94-fold at moi 100) (Fig. 3a). The infectivity of AdΔCAR-LucEGFP-PFW was lower than AdΔCAR-LucEGFP-SYE in AsPC-1 cells in culture (Fig. 3a), whereas AdΔCAR-LucEGFP-PFW showed a higher luciferase activity compared with AdΔCAR-LucEGFP-SYE in AsPC-1 peritoneal tumors (Fig. 2b). The results suggest that the *in vivo* screening is also a promising strategy in developing the targeted adenovirus vectors.

To validate that the targeting is mediated by the selected peptide, the transduction efficiency of AdΔCAR-LucEGFP-PFW in AsPC-1 cells was determined in the presence or absence of the cognate peptide. A substantial competitive inhibition by the cognate peptide but not by a control unrelated peptide was observed at moi 30 in a dose-dependent manner (Fig. 3b), confirming that the enhanced transduction of the targeted vector in the cells is mediated by the insertion of the selected peptide.

### Infectivity of Adenovirus Displaying a Selected Peptide for Other Peritoneal Tumors and Organs

**Figure 4:**
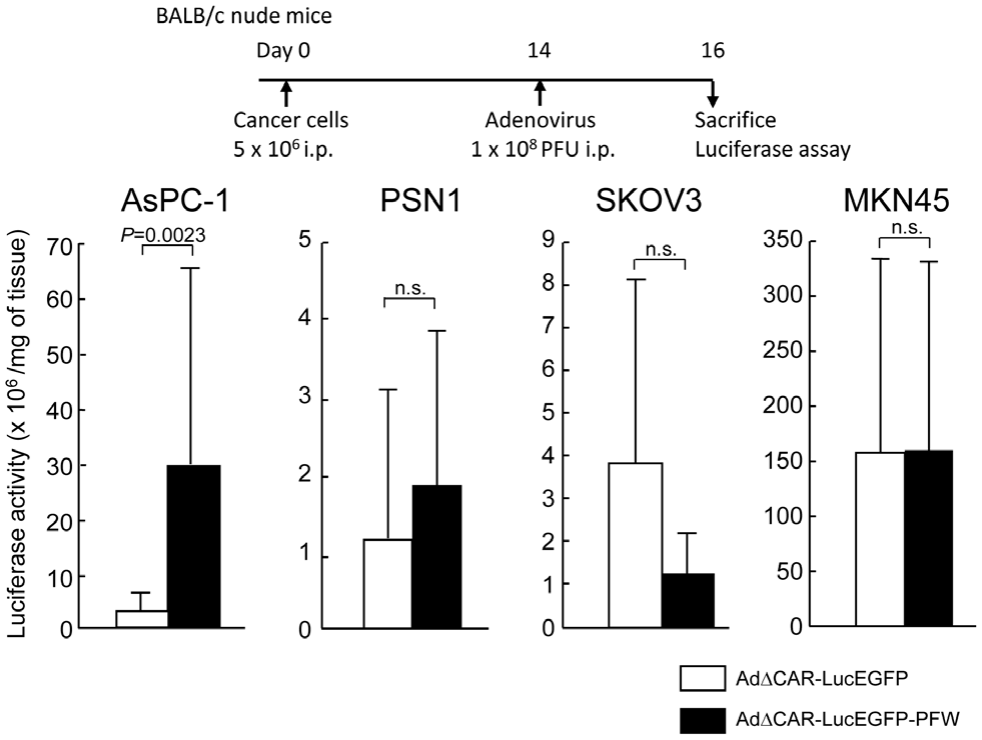
Infectivity of various peritoneal tumors with the adenovirus displaying the selected peptide. The mice were injected intraperitoneally with 1×10^8^ PFU of adenoviruses (AdΔCAR-LucEGFP-PFW or AdΔCAR-LucEGFP) (AsPC-1; n = 14, others; n = 4), and 2 days after the injection, peritoneal tumors were harvested and the luciferase activity was measured.

To examine whether the adenovirus vector displaying the selected peptide could effectively transduce the gene in various peritoneal tumors, the luciferase activity was assessed in AsPC-1, PSN-1, SKOV3 and MKN45 peritoneal tumors 2 days after the intraperitoneal injection of the vectors. The AdΔCAR-LucEGFP-PFW showed a higher luciferase activity in AsPC-1 peritoneal tumors than did AdΔCAR-LucEGFP as shown in Fig. 2a, whereas the luciferase activities in 3 other tumors of AdΔCAR-LucEGFP-PFW-injected mice were not significantly elevated compared with those of AdΔCAR-LucEGFP-injected mice (PSN-1; 1.6-fold, SKOV3; 0.32-fold, MKN45; 1.0-fold) (Fig. 4), suggesting the specificity of a selected peptide for AsPC-1 peritoneal tumors. Although the AdΔCAR-LucEGFP-PFW showed different infectivity between *in vitro* culture and peritoneal tumors for AsPC-1 cells, its infectivity in peritoneal tumors was compatible with those in *in vitro* culture for PSN-1, SKOV3 and MKN45 cells (Figs. 3a and 4), demonstrating the importance of *in vivo* screening with target cancer cells.

**Figure 5:**
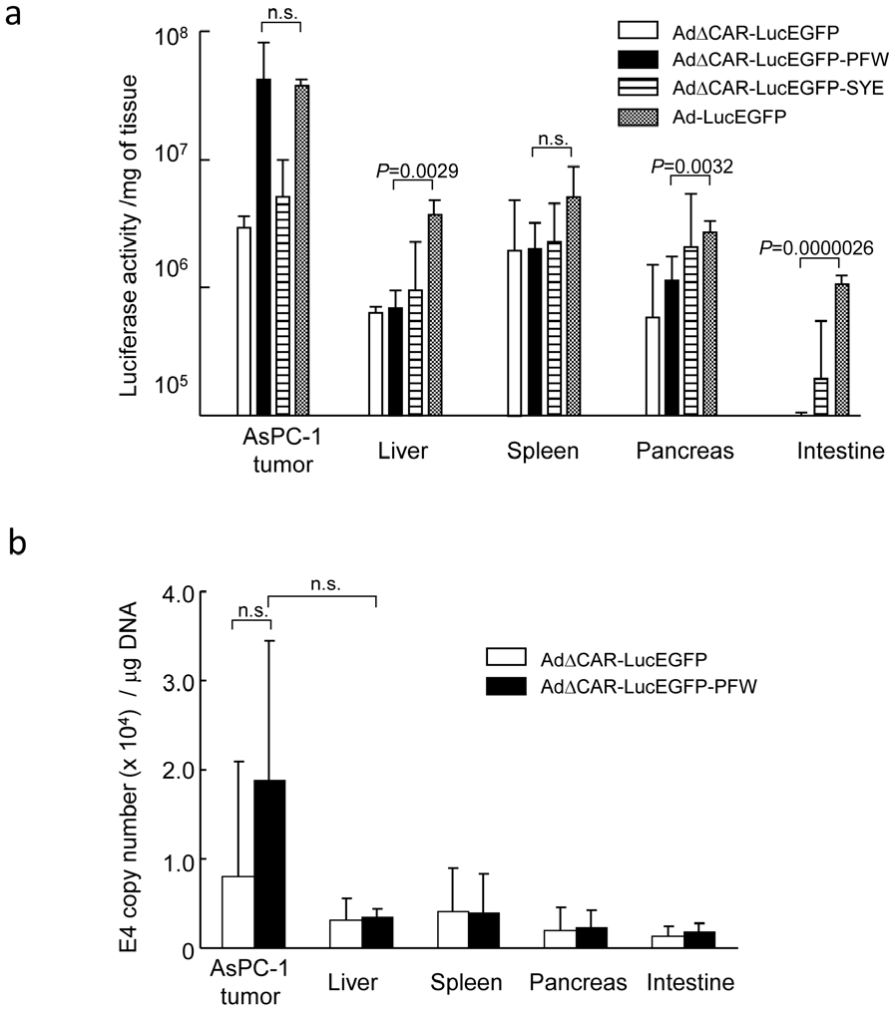
Infectivity of organs with the adenovirus displaying the selected peptide. (a) Infectivity of organs with adenovirus vectors. The mice were injected intraperitoneally with 1×10^8^ PFU of adenovirus vectors (AdΔCAR-LucEGFP-PFW, AdΔCAR-LucEGFP-SYE, AdΔCAR-LucEGFP or Ad-LucEGFP)(n = 3∼5), and 2 days after the injection, organs such as the liver, spleen, pancreas and small intestine were harvested and the luciferase activities were measured. (b) Distribution of adenovirus DNA after intraperitoneal injection of adenovirus vectors. DNAs from AsPC-1 tumors and organs (n = 3) were analyzed by a real-time quantitative PCR for the adenovirus E4 copy number. The results are shown as adenoviral copy number per 1 µg of tissue DNA.

Finally, to confirm the AsPC-1 tumor-targeting effect of the selected vector, various organs such as liver, spleen, pancreas and small intestine were also harvested 2 days after the intraperitoneal injection of vectors at a dose of 1×10^8^ PFU. The luciferase activities in the organs were markedly lower than those in AsPC-1 tumors in the AdΔCAR-LucEGFP-PFW-injected mice. The luciferase activities in organs of AdΔCAR-LucEGFP-PFW-injected mice were generally similar to those of AdΔCAR-LucEGFP-SYE- and AdΔCAR-LucEGFP-injected mice, which were lower than those of Ad-LucEGFP-injected mice (Fig. 5a), indicating that the selected peptide does not enhance the infectivity in the organs.

Furthermore, we analyzed the distribution of adenovirus DNA by a real-time quantitative PCR method in peritoneal tumors and various organs 2 days after the injection of adenovirus vectors. Although the quantitative PCR showed that the adenovirus DNA was more detected in the peritoneal tumors when compared with organs in AdΔCAR-LucEGFP-PFW-injected mice, the difference was not statistically significant (Fig. 5b). Considering that the luciferase assay showed much lower expression of the gene in organs than in peritoneal tumors (Fig. 5a), it is plausible that DNA detected by quantitative PCR analysis might be the partially degraded fragments of adenoviral DNA.

## Discussion

The redirection of virus tropism is one of the most rational approaches to targeted vector development. We have shown that the *in vitro* screening of a random peptide display adenovirus library on a cell type of interest allows the selection of targeted adenovirus vectors as previously reported [14], [15]. Here, we screened pancreatic peritoneal dissemination with the adenovirus library and identified a candidate targeting ligand sequence, which was not isolated from the same library by *in vitro* screening on AsPC-1 cells. The *in vivo* library screening is also useful to explore the targeting vectors as well as screening on culture cells, and it may be important to employ the experimental models in accordance with an intended disease condition of target cancer cells in the library screening for developing a targeted vector.

The infectivity of AdΔCAR-LucEGFP-PFW for AsPC-1 peritoneal tumors was similar with that of Ad-LucEGFP, whereas the infectivity of AdΔCAR-LucEGFP-PFW for organs was much lower than Ad-LucEGFP (Fig. 5a). The relative luciferase activity of AdΔCAR-LucEGFP-PFW for peritoneal tumor compared with the liver was 61.5-fold, whereas that of Ad-LucEGFP was 9.8-fold, demonstrating that the insertion of PFWSGAV in the fiber knob enhanced the *in vivo* tumor-specificity of adenovirus vector. The results suggest that the combination of a targeting peptide with suppression of naïve viral tropism improves the safety compared with the wild type of fiber. In case we do not need to consider the infection of adenovirus vectors in organs, the incorporation of targeting peptides into the capsid of wild fiber may be useful to more enhance the infectivity for target tumors.

In our previous manuscript, we showed that approximately 10% of clones in the shuttle plasmid libraries converted successfully into adenovirus virions, whereas 90% of the peptide insertions into the HI-loop disturbed virus production, possibly due to the conformational change of the fiber and disturbance of the fiber trimerization [14]. Therefore, we estimate that the maximum complexity of the 7 amino acids displayed on the fiber knob is at the1×10^8^ level. The 2×10^5^ complexity level used in this study represents only a small fraction of the possible vector clones. Although the selected peptide would be the most efficient sequence in this adenovirus library, more efficient peptide motifs can be isolated by a scaling up of the library size. Since the complexity of an adenovirus library depends on the number of helper cells transfected with recombined DNA, the simple scaling up of the number and size of the plates could increase the complexity of the library. We intend to employ a high complexity of library in comprehensively exploring targeted vectors for various tumors.

A CAR-binding region in the backbone adenovirus construct is ablated to reduce the natural tropism. However, since CAR-binding ablation alone does not strongly reduce the *in vivo* natural tropism of the adenovirus vector, the additional ablation of binding sites with integrin and heparan sulfate proteoglycans from the adenoviral capsid should be useful to further reduce naïve tropism [21]. Furthermore, it was reported that coagulation factor (F) X directly binds the hypervariable regions (HVR) of the hexon surface in an adenovirus, leading to liver infection [22]. A targeted adenovirus constructed on a mutant HVR backbone to suppress a liver transduction might effectively allow for the development of vectors to specifically transduce certain tumors even through systemic administration.

Although there was a possibility to isolate peptide sequences that can generally enhance gene transduction in various tumors and organs, a selected peptide showed AsPC-1 peritoneal tumor type-directed infection (Figs. 4 and 5a), which was consistent with our previous *in vitro* results [14], [15], suggesting that library screening based on the binding to target tumor cells may enable identification of tumor specific-targeting ligands. However, it may be useful to incorporate a negative selection method in the early screening phase in addition to the positive selection. We are developing 2 strategies for negative selection: one is the absorption of vectors, which show high infectivity for many cells, upon the mixture of various normal cells before the screening on the target cells. The other strategy is *in vivo* systemic screening of the library to specifically transduce tumors using animal models, since normal tissues may absorb the vectors with generally high infectivity. In fact, Wu et al. isolated an adenovirus vector targeting to prostate-specific membrane antigen (PSMA) through virus-displayed semirandom peptide display screening by counter and positive selections with PSMA-expressing cancer cells and a systemic injection in a tumor mouse model [23].

A substantial competitive inhibition of AdΔCAR-LucEGFP-PFW by the cognate peptide suggests that the infection of targeted vectors was mediated by receptors on target cells. The corresponding target receptor of the isolated ligand may be a specific cell surface molecule or its expression may be significantly higher on the AsPC-1 tumors. The identification of the receptors would be useful to understand the molecular characteristics of tumors and can be applied for diagnosis, such as the detection of a relapse, and for therapy of the disease. However, database searches (BLAST) did not reveal sequence homology of the selected peptide with known human proteins. Thus, the candidate receptor responsible for the PFWSGAV-mediated infection in AsPC-1 tumors is unknown. Additional work is necessary to identify the corresponding receptors.

Recently, promising preclinical and clinical data on the treatment of cancer using a conditionally replication-competent adenovirus (CRAd) have been reported [24], [25]. In this study, we screened a peptide-display adenovirus library on pancreatic tumors to explore targeting ligands for pancreatic peritoneal dissemination, which is often recognized at advanced stages and may be a clinical target of local oncolytic virus therapy. Although the infection and spreading of such CRAds are restricted to tumor tissue in theory, several levels of safety devices are definitely required in the clinical setting. Therefore, the combination of CRAds with target specificity is a highly encouraging strategy for gene therapy. Furthermore, we previously reported that the intratumoral injection of a replication-competent adenovirus displaying a selected ligand showed a higher oncolytic potency when compared with the untargeted adenovirus vector in subcutaneous tumor models [15]. The antitumor effect of an oncolytic virus is determined by the capacity to infect tumor cells [26], [27]. Therefore, we expect that the insertion of the PFWSGAV sequence into the replication-competent adenovirus vector could enhance the oncolytic activity for AsPC-1 peritoneal tumors. A library approach with a replication-competent adenovirus may be highly useful to isolate a targeted oncolytic adenovirus, because the most efficient adenovirus should be selected from the library based on its high infectivity and replication capacity through the process of virus amplification and its spread through target tumors.

Future development will be a screening on the biopsy and surgical materials of the tumor, thereby leading to a generation of individualized targeted viruses. On the other hand, extensive exploration of target peptides with the *in vitro* and *in vivo* screening of an adenovirus library may allow the making of a list of targeting peptides for each cancer, and it may be more convenient to select a targeted vector suitable for an individual patient from the list. This library-based technology for a specific adenovirus vector selection may have broad implications for a variety of applications in medicine and medical sciences.
